# Development of a genetically programed vanillin-sensing bacterium for high-throughput screening of lignin-degrading enzyme libraries

**DOI:** 10.1186/s13068-017-0720-5

**Published:** 2017-02-03

**Authors:** Barindra Sana, Kuan Hui Burton Chia, Sarada S. Raghavan, Balamurugan Ramalingam, Niranjan Nagarajan, Jayasree Seayad, Farid J. Ghadessy

**Affiliations:** 1 p53 Laboratory, Agency for Science Technology And Research (A*STAR), 8A Biomedical Grove, #06-04/05 Neuros/Immunos, Singapore, 138648 Singapore; 2 Genome Institute of Singapore, 60 Biopolis Street, Genome, #02-01, Singapore, 138672 Singapore; 3 Institute of Chemical and Engineering Sciences, 8 Biomedical Grove, Neuros, #07-01, Singapore, 138665 Singapore

**Keywords:** Directed evolution, Fluorescence-activated cell sorting, RNA sequencing, Lignin, Vanillin, Enzyme engineering, Biorefinery, Vanillin-inducible promoter, Microbial biosensor, High-throughput screening

## Abstract

**Background:**

Lignin is a potential biorefinery feedstock for the production of value-added chemicals including vanillin. A huge amount of lignin is produced as a by-product of the paper industry, while cellulosic components of plant biomass are utilized for the production of paper pulp. In spite of vast potential, lignin remains the least exploited component of plant biomass due to its extremely complex and heterogenous structure. Several enzymes have been reported to have lignin-degrading properties and could be potentially used in lignin biorefining if their catalytic properties could be improved by enzyme engineering. The much needed improvement of lignin-degrading enzymes by high-throughput selection techniques such as directed evolution is currently limited, as robust methods for detecting the conversion of lignin to desired small molecules are not available.

**Results:**

We identified a vanillin-inducible promoter by RNAseq analysis of *Escherichia coli* cells treated with a sublethal dose of vanillin and developed a genetically programmed vanillin-sensing cell by placing the ‘very green fluorescent protein’ gene under the control of this promoter. Fluorescence of the biosensing cell is enhanced significantly when grown in the presence of vanillin and is readily visualized by fluorescence microscopy. The use of fluorescence-activated cell sorting analysis further enhances the sensitivity, enabling dose-dependent detection of as low as 200 µM vanillin. The biosensor is highly specific to vanillin and no major response is elicited by the presence of lignin, lignin model compound, DMSO, vanillin analogues or non-specific toxic chemicals.

**Conclusions:**

We developed an engineered *E. coli* cell that can detect vanillin at a concentration as low as 200 µM. The vanillin-sensing cell did not show cross-reactivity towards lignin or major lignin degradation products including vanillin analogues. This engineered *E. coli* cell could potentially be used as a host cell for screening lignin-degrading enzymes that can convert lignin to vanillin.

**Electronic supplementary material:**

The online version of this article (doi:10.1186/s13068-017-0720-5) contains supplementary material, which is available to authorized users.

## Background

Plant biomass is a potential renewable raw material for sustainable production of biofuels and value-added chemicals. The three major constituents of plant biomass are cellulose (40–43%), hemicellulose (20–27%) and lignin (20–30%). Huge amounts of lignin are produced as a by-product of the paper industry, while cellulosic components of plant biomass are utilized for the production of paper pulp. In the near future, biorefineries will generate substantial amounts of lignin by-products after converting plant cellulose to bioethanol, which have no significant use apart from burning for energy. Compared to cellulose, lignin has extremely heterogeneous aromatic building blocks that can potentially be converted into various value-added chemicals or precursors for the synthesis of commodity chemicals. Lignin could serve as a potential source of aromatics that can substitute fossil-derived consumer products [1–3].

In spite of its vast potential, lignin remains the least exploited component of plant biomass due to its recalcitrant nature that is attributed to the extremely complex cross-linked three-dimensional structures of the lignin backbone [4, 5]. Vanillin is the most lucrative lignin degradation product due to its higher cost and notable demand in the food, flavour and cosmetics industries. Other lignin degradation products like acetovanillone, vanillyl alcohol, syringaldehyde, guaiacol and eugenol also have potential industrial applications. Although once a common industrial practice, chemical conversion of lignin to vanillin is not widely used today due to hazardous environmental impacts of chemical conversion methods. Only one major company is still producing vanillin from spent sulfite liquor by a chemical process [6, 7]. The search for greener alternatives is leading to the development of chemical catalysts that can potentially lead to oxidative lignin degradation under mild conditions [8–10]. Biocatalysts may play an important role as several microorganisms are well known for recycling abundant lignin biomass in nature and a few natural enzymes have been reported with lignin-degrading properties [11–17]. Reiter et al. demonstrated depolymerization of complex lignin into small amounts of aromatic monomeric compounds using a combination of Cα-dehydrogenase, β-etherase and glutathione lyase enzymes [15]. Studies suggest the production of small phenolic compounds (acids, ketones and aldehydes) via oxidative lignin degradation by white-rot fungi [18–21]. Very recently, Salvachúa et al. [22] have reported the partial depolymerization of high-lignin content biorefinery stream using fungal secretomes containing high laccase and peroxidase activity in the presence of an aromatic-catabolic bacterium as a ‘microbial sink’. However, to date no enzyme is reported to degrade lignin to the monomeric phenolic subunits with high efficiency. Development of robust lignin-degrading enzymes by engineering the catalytic efficiency of currently available enzymes would be a valuable step forward in implementing white biotechnology processes for the conversion of lignin biomass to value-added chemicals.

Directed evolution is a protein engineering technique whereby extremely large numbers (up to 10^10^) of mutant enzymes are generated and rapidly screened for the desired characteristics [23–25]. However, the application of this technique for developing effective lignin-degrading enzymes is limited due to the lack of an efficient high-throughput screening method that is essential for rapid screening of large numbers of mutants. Several reports have described the directed evolution of lignin-degrading enzymes such as laccase and peroxidase using plate-based colorimetric screening methods [26–29]. These strategies have been useful in generating enzymes with desired characteristics such as higher redox potential, improved expression level, altered substrate specificity and organic solvent tolerance. However, these studies have mainly employed readily oxidizable colorimetric proxy substrates such as 2,2′-azino-bis(3-ethylbenzothiazoline-6-sulphonic acid) (ABTS), which does not necessarily result in these enzymes showing a lignin-degrading phenotype. Direct detection of lignin degradation products is the ideal way to identify efficient lignin-degrading enzymes generated by directed evolution, but current product detection methods of choice such as GC/MS and LC/MS are time consuming and not suitable for high-throughput enzyme screening. Development of a biosensor that can detect a lignin degradation product by transducing the metabolite concentration to reporter gene expression would be useful in rapid phenotypic evaluation of specific product formation and identification of superior enzyme variants within an engineered enzyme library.

**Fig. 1:**
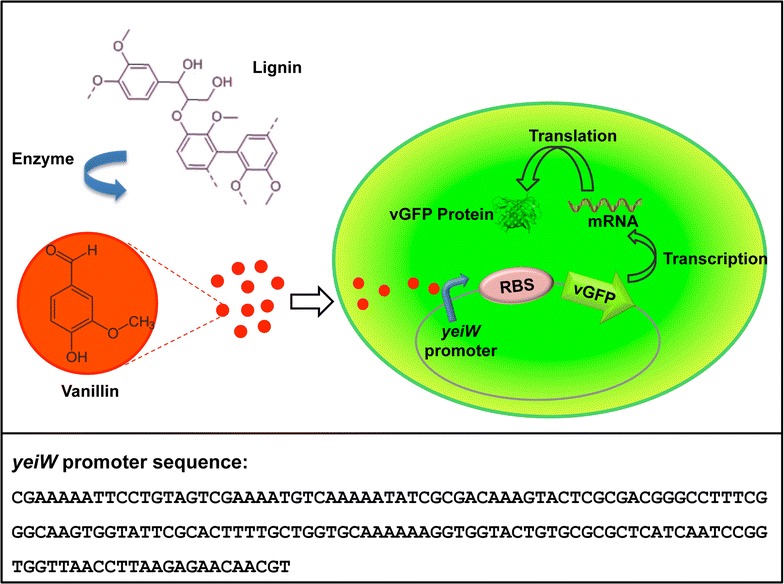
Schematic diagram of the vanillin-sensing cell (VSC). The reporter plasmid construct comprises the vGFP gene cloned downstream of the vanillin-inducible promoter *yeiW*. The presence of vanillin results in a readily detectable fluorescence phenotype. For enzyme screening applications, any vanillin generated through extracellular degradation of lignin would activate the genetically encoded biosensor

Inducible regulator-based systems for gene expression are regulated by the presence of a specific small molecule inducer. The IPTG-inducible *LacI* promoter is the prototypical example of a small-molecule-inducible system and is widely used in hyper-expression of recombinant genes. The engineered *LacI* promoter-based system has also been used in signal processing and chromosomal visualization [30, 31]. If a reporter gene is controlled by this inducible regulator, the presence of the inducing molecule can be detected by the phenotypic change due to the production of the reporter protein. While a few well-characterized small-molecule-inducible regulators (LacI, AraC, TetR etc.) are widely used in several applications, the development of additional inducible systems will expand their use in innovative areas of biotechnology, such as metabolic engineering. Detection of a target chemical using live cell biosensors would be a robust technique for directed evolution methodologies. Such small-molecule-inducible biosensors could be developed using DNA constructs that control the expression of a reporter gene in response to the presence of the specific target molecule. The utility of inducible microbial biosensors was recently demonstrated through monitoring glucarate production in a heterologous glucarate biosynthesis pathway and identification of superior enzyme variants using a live cell glucarate sensor [32]. This study suggests the potential of small-molecule-inducible biosensors in screening enzyme libraries for the production of the inducing chemicals. Here, we describe the development and characterization of an inducible whole-cell biosensing system (i.e. an engineered *E. coli* cell) that can detect the presence of vanillin, a commercially attractive lignin degradation product (Fig. 1). This biosensor has potential use in the screening of engineered enzymes that could convert lignin to vanillin.

## Methods

### Chemicals and reagents

All chemicals including kraft lignin, vanillin, vanillin analogues, benzaldehyde, DMSO and acrylic acid used in this study were purchased from Sigma-Aldrich (USA). The lignin model compound (guaiacylglycerol-beta-guaiacyl ether) was from Tokyo Chemical Industries Co., Ltd. (Japan).

### Identification of up-regulated genes by RNAseq analysis

#### Vanillin treatment and RNA isolation

*Escherichia coli* BL21 cells were cultured in LB medium at 37 °C and exposed to increasing concentration of vanillin at the mid-log phase (OD_600_ = 0.5), and the sublethal dose of vanillin was determined from the growth curves. For RNAseq experiments, fresh *E. coli* BL21 culture was exposed to 0 and 5 mM vanillin (sublethal concentration) at the mid-log phase and the cells were collected at 0, 1, 2 and 3 h post exposure. Total RNA was extracted from the *E. coli* cells using RNeasy Mini kit (Qiagen) following the manufacturer’s protocol, and rRNA was removed using ribo-zero rRNA removal kit (Epicentre) following the manufacturer’s protocol. The experiment was done in triplicate, the RNA was quantified and quality was confirmed from the high RIN values (>9.0) in the Bioanalyzer quality analysis.

#### Gene-based expression matrix

A gene-based expression matrix was generated from the BAM files using Cuffnorm v2.2.0, a program that is part of Cufflinks [33]. Cuffnorm was run with the options of “–library-type fr-unstranded” and “–library-norm-method classic-fpkm”. The resulting expression matrix is normalized for library size and the values are represented as FPKM (fragments per kilobase of exon per million fragments mapped).

#### Hierarchical clustering

Hierarchical clustering was performed on the FPKM expression matrix in R v3.1.0. The expression matrix was first transformed to the log-2 space before computing the distance matrix based on the Euclidean distance of measure using the dist function of R. The Spearman correlation was then calculated using the cor function before being plotted using the heatmap.2 function from the gplots package from CRAN.

#### Differential expression analysis

Cuffdiff v2.1.1, a program that is part of Cufflinks, was used to identify differentially expressed genes at each time point [33]. Default parameters were used except for the option of “–multi-read-correct” and “–max-bundle-frags 100000000”. A threshold of FDR < 0.05 and absolute fold change >2.0 were used for significance. SAM (Significance Analysis of Microarrays) was used to identify genes that were differentially expressed across time points. This analysis was performed in R v3.1.0 using the samr package from CRAN with the following options: resp.type = “Two class unpaired timecourse”, nperms = 100 and time.summary.type = “slope”. Genes having a log-2 fold change >2.0 were identified as up-regulated, while those having a log-2 fold change smaller than −2 were identified as down-regulated.

### Plasmid construction and biosensor development

#### Prediction of putative promoter regions and plasmid construction

The putative promoter regions of the top seven up-regulated genes were arbitrarily predicted to be located within the first 300 bp of the non-coding region immediately upstream of the up-regulated genes (Additional file 1: Tables S1, S2). The putative promoters were amplified by PCR using suitable infusion cloning primers. The amplified products were cloned upstream of the very green fluorescence protein (vGFP) gene [34] in a customized plasmid construct developed in pUC19 backbone by replacing ~600-bp nucleotides after the origin of replication (including the lac promoter sequence) by the vGFP gene, using Infusion HD cloning kit (Clontech Laboratories). The predicted endogenous ribosome binding sites (RBS) were replaced by a strong g10 RBS sequence ‘tttaactttaagaaggagatatacat’ [32]. The final plasmid constructs contain an ampicillin resistance gene, *E. coli* origin of replication and a vanillin-inducible putative promoter region followed by the RBS and the vGFP gene (Fig. 1; Additional file 1: Figure S3).

#### Biosensor development and selection

Seven live cell biosensors (Lcb1–Lcb7) were developed by transforming chemically competent *E. coli* BL21 cells with seven plasmid constructs containing different putative promoter sequences (Fig. 1; Additional file 1: Table S2). The live cell biosensors were grown in LB medium at 37 °C up to mid-log phase followed by overnight induction with 5 mM vanillin. The 5 mM final vanillin concentration was achieved by 400 times dilution of 2.0 M stock solution in DMSO; a set of control experiments was done without the addition of vanillin but in the presence of an equivalent amount of DMSO. The cells were collected by centrifugation, washed with phosphate-buffered saline (PBS) and resuspended in the same buffer to make the cell concentration to OD_600_ = 1. Expression of the vGFP was estimated by measuring green fluorescence (Ex/Em = 488/509 nm) of 100 µl resuspended cells (OD_600_ = 1.0) using a multilabel plate reader (PerkinElmer 2104), and the level of induction was calculated from the fluorescence ratio of induced to uninduced cells of each live cell biosensor. High induction level of the best biosensor (with the highest increase of fluorescence) was confirmed by measuring the green fluorescence of induced and uninduced cells by fluorescence-activated cell sorting (FACS) analysis using BD FACSAria cell sorter (BD Biosciences) and the sensor was selected for further characterization.

### Characterization of the selected live cell biosensor

#### Sensitivity of the live cell biosensor to vanillin concentration

The relationship between vanillin concentration and the expression of the fluorescent reporter was evaluated. 1 ml overnight culture of the live cell biosensor was inoculated in 100 ml LB medium and cultured at 37 °C with constant shaking at 175 rpm until the OD_600_ reached 0.5. Then the culture was split into twelve 5-ml portions and induced (in duplicate) for 20 h with 0, 0.2, 0.5, 1.0, 3.0 and 5.0 mM vanillin. The cells were collected by centrifugation, washed with phosphate-buffered saline (PBS) and resuspended in the same buffer. A portion of the cell suspension was further diluted to prepare a 2-ml sample with a final cell concentration of OD_600_ = 0.1. Response of the live cell biosensor to various vanillin concentrations was estimated from median fluorescence of the cells measured by FACS analysis using a BD FACSAria cell sorter (BD Biosciences). The experiment was repeated three times independently and the average fluorescence of the biosensor treated with individual vanillin doses was calculated after normalizing fluorescence of the control to 100.

#### Cross-reactivity testing

Cross-reactivity of the live cell biosensor was tested against a panel of potential inducing compounds including various lignin degradation products (acetosyringone, acetovanillone, guaiacol, syringaldehyde, vanillic acid and vanillyl alcohol), kraft lignin, dimeric lignin model compound (guaiacylglycerol-beta-guaiacyl ether), DMSO, benzaldehyde, veratraldehyde and a non-specific toxic chemical (acrylic acid). The live cell biosensor was grown to mid-log phase and treated with three different concentrations of each chemical individually. The cultures were grown for 20 h and cells were collected by centrifugation. To study its performance in real lignin-degrading condition, the biosensor was also treated with two mixtures: (1) 5 mg/ml alkaline kraft lignin and 5 mM vanillin and (2) 5 mg/ml alkaline kraft lignin and 5 mM each of acetosyringone, acetovanillone, guaiacol, syringaldehyde, vanillyl alcohol and vanillin. Cross-reactivity of the biosensor to the individual chemicals and their mixtures was assessed from the fluorescence of the cells measured by FACS analysis of the samples after washing and diluting with PBS. The experiment was repeated three times independently in duplicate, and the average fluorescence of the vanillin-sensing cells treated with individual chemicals was calculated after normalizing fluorescence of the control to 100.

#### Fluorescence microscopy

Increased fluorescence of the vanillin-induced live cell biosensor was visualized under a fluorescence microscope and compared with the fluorescence of untreated and non-specific chemical-treated biosensor. The biosensor was treated with 5 mM vanillin, guaiacol, acrylic acid or 5 mg/ml lignin and grown for 20 h; one control is prepared without treatment with any chemical. The cells were washed with PBS and diluted to OD_600_ = 1.0. One drop of the cell suspension was placed on a microscope slide, air dried and covered with a coverslip. All samples were observed under the AxioImager Z1 upright fluorescent microscope (Zeiss) using 63× oil immersion lense and imaged with 500 ms exposure time. Mean intensity of the cells was measured using Fiji software.

#### Toxicity test

Toxicity of various lignin degradation products and the non-specific chemical acrylic acid was determined by growing the live cell biosensor in the presence of various concentrations of each chemical. LB medium containing 0.1 mg/ml ampicillin was inoculated with 1% (v/v) overnight culture of the live cell biosensor. 2 M stock solutions of acrylic acid (in water) and the lignin degradation products including vanillin, vanillic acid, vanillyl alcohol, syringaldehyde, guaiacol, acetovanillone and acetosyringone (in DMSO) were added immediately to obtain the final concentrations of 2.5, 5.0, 10.0 and 20.0 mM. A control was prepared without the addition of any chemical. The cells were grown at 37 °C with constant shaking at 175 rpm and growth was monitored by measuring OD_600_ at regular time intervals. The experiment was repeated three times independently and the growth curves were obtained by plotting average cell density against time. Toxicity of the chemicals at each concentration was estimated by comparing the growth curve with the control in which no chemical was added.

## Results

Vanillin is toxic to *E. coli* at high concentrations [35]. Treatment with vanillin showed that the growth of *E. coli* was significantly inhibited at concentrations ≥5 mM and that the cells started to recover 2.5 h post treatment with 5 mM vanillin (Additional file 1: Figure S1). To identify genes that are regulated to mediate the response to vanillin exposure, we carried out RNAseq analysis of *E. coli* cells treated with 5 mM vanillin [36]. Significant variations in global gene expression profiles were observed between vanillin-treated and control samples collected at different time points for the RNAseq experiment (Additional file 1: Figure S2). Differentially expressed genes were further identified by comparing RNA levels of vanillin-treated cells with those of untreated cells at individual time points. These identified 759 *E. coli* genes that were differentially expressed across all time points, of which 725 genes were down-regulated and 34 genes were up-regulated. There was no clear functional clustering of the up- or down-regulated genes. Several genes encoding inner membrane proteins such as *ygbE*, *mrp*, *ydjX* and *yjgN* were down-regulated by vanillin treatment; however, their precise functions remain unknown. Analysis of the top seven up-regulated genes suggests their association in multiple physiological functions including osmoprotection, stress response and heavy metal detoxification (Additional file 1: Tables S1). The top two up-regulated genes *yjhD* and *yijF* have unknown functions, while the three up-regulated genes *ydcI*, *yeiW* and *sodC* potentially contribute to heavy metal detoxification and oxidative stress defence. The fourth highest up-regulated gene *proA* is involved in the biosynthesis of the osmoprotective amino acid proline, and the other up-regulated gene *higA* encodes an antitoxin of the HigB–HigA toxin–antitoxin system. Locations of the top seven up-regulated genes within the *E. coli* genome and their upstream/downstream sequences were manually investigated (Additional file 1: Table S1). Promoter regions were arbitrarily predicted to be located within the first 300 bp of the non-coding region immediately upstream of the up-regulated genes. The putative promoter regions from these top seven up-regulated genes (Additional file 1: Table S2) were cloned individually upstream of the vGFP gene in a customized plasmid, and seven live cell biosensors (Lcb1–Lcb7) were generated by transforming *E. coli* BL21 cells with these plasmid constructs (Fig. 1; Additional file 1: Figure S3). The vGFP gene of each biosensor was overexpressed by inducing with 5 mM vanillin and the production of the vGFP protein was estimated from the fluorescence of the overnight induced cells (Additional file 1: Table S3). The sensors Lcb4 and Lcb5 showed high levels of fluorescence in the presence of vanillin although uninduced cells also showed background fluorescence due to leaky nature of the promoters. Fold induction of these biosensors was calculated from the fluorescence ratio of induced to uninduced biosensors measured by FACS analysis. The biosensor Lcb5 constructed with the putative promoter region upstream of the *yeiW* gene showed higher fluorescence enhancement (4.3 fold) compared to Lcb4 that was made with the putative promoter region upstream of the *proA* gene. Lcb4 showed high background fluorescence with 1.8-fold fluorescence enhancement. The biosensor Lcb5 (hereafter termed ‘vanillin-sensing cell’ or VSC biosensor) was further characterized for sensitivity to vanillin, cross-reactivity and toxicity towards lignin, lignin model compounds, solvents, potential lignin degradation products, vanillin analogues and non-specific toxic chemicals.

### Sensitivity of VSC biosensor to vanillin

**Fig. 2:**
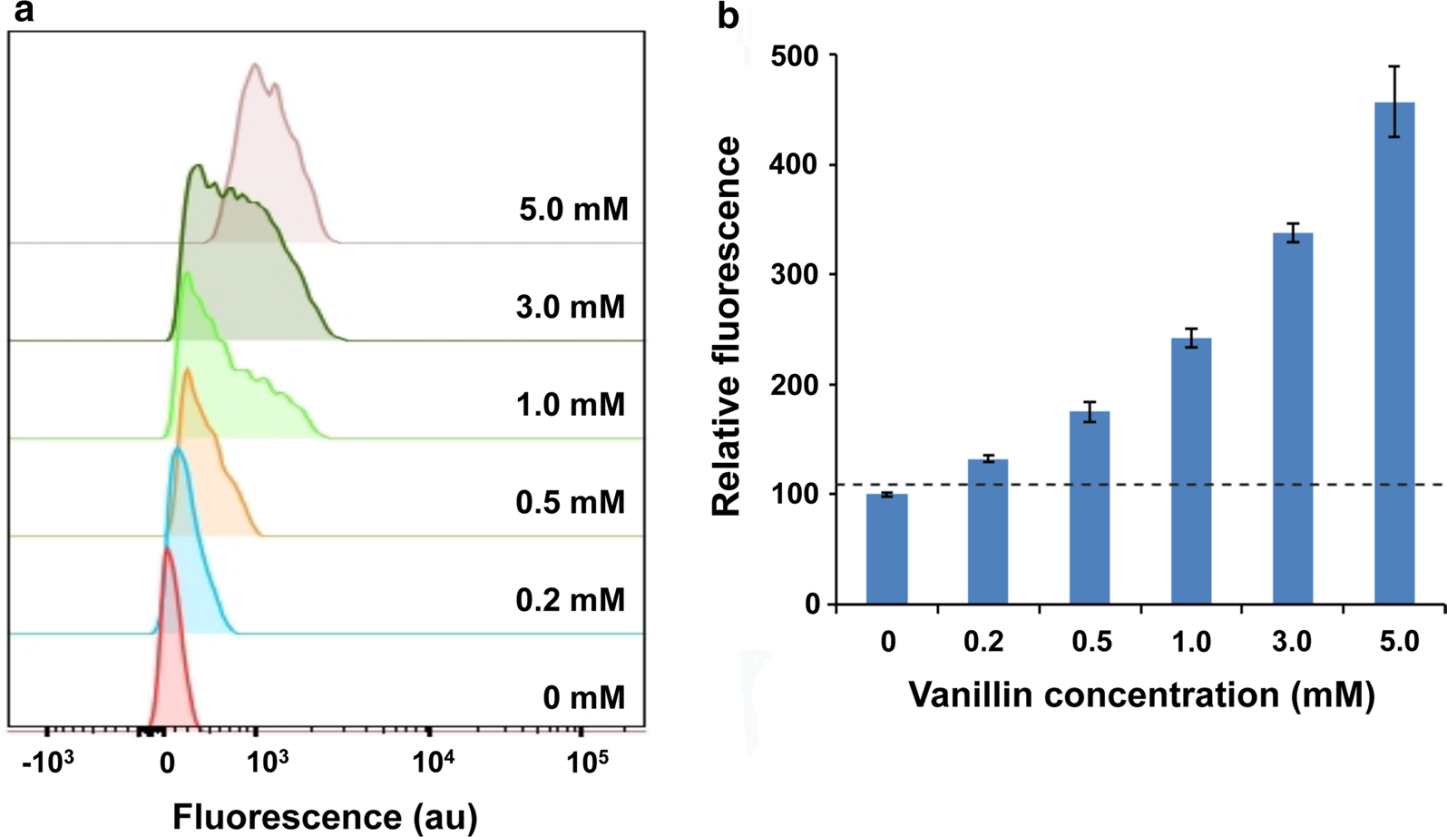
Response of the VSC biosensor to various vanillin concentrations: **a** histogram of FACS fluorescence measurements and **b** median fluorescence value (normalized) of the populations in each sample. Biosensing cells were treated with indicated vanillin concentrations and analysed by FACS. The *dotted line* in the column chart indicates the significance threshold (fluorescence of the untreated cells + 3 SD)

**Fig. 3:**
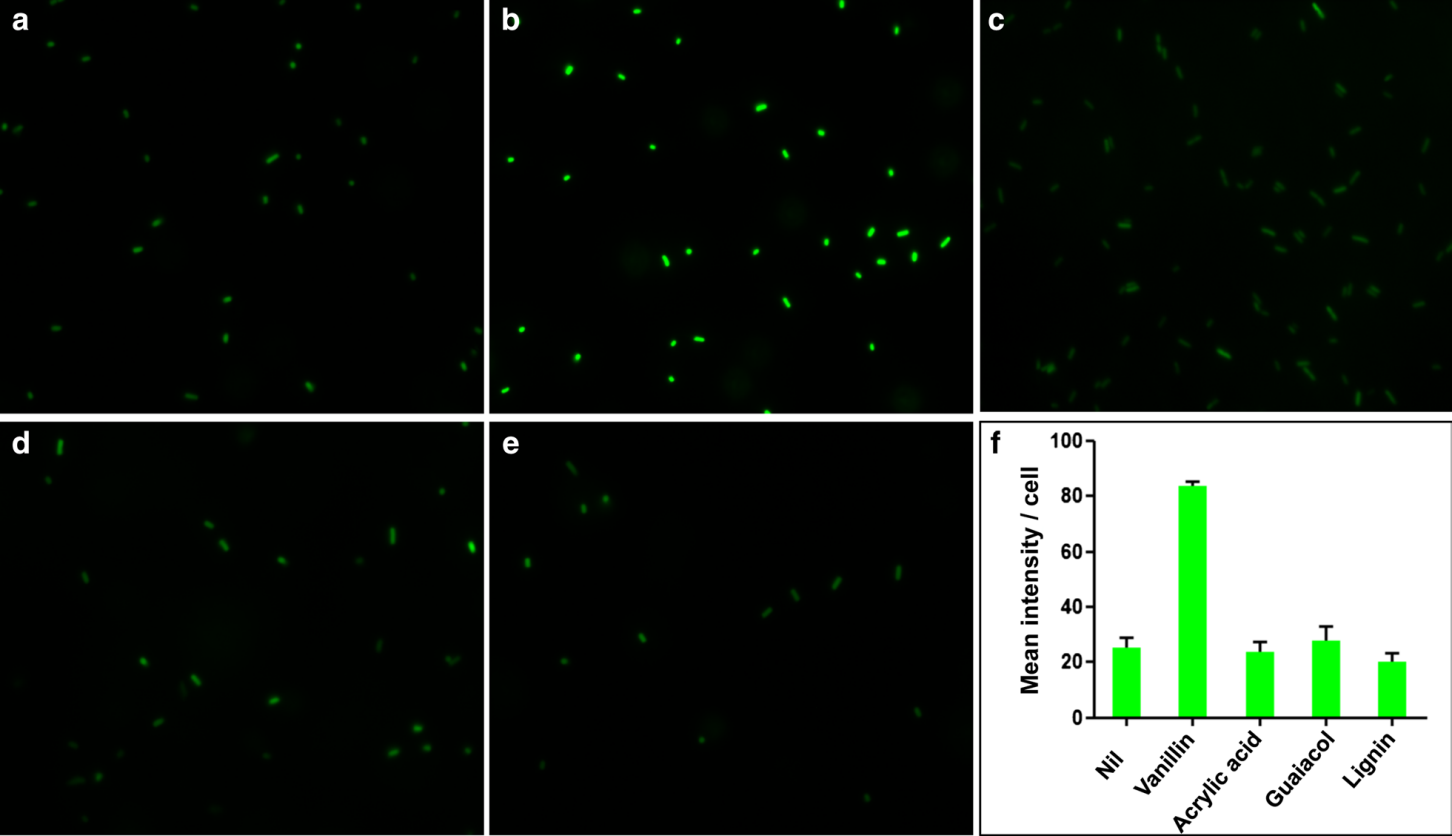
Fluorescence microscopy image of the VSC biosensor: **a** untreated and after treatment with 5 mM, **b** vanillin, **c** acrylic acid, **d** guaiacol and **e** 5 mg/ml lignin. Mean fluorescence of the cells was measured using ImageJ software and is presented in **f**. The cells were grown for 20 h in the presence of individual chemicals, and all the samples were imaged by fluorescence microscopy using same magnification and exposure times

The VSC biosensor was then tested against different concentrations of vanillin to determine its detection threshold. The biosensor responds to different concentrations of vanillin in a dose-dependent manner (Fig. 2). FACS analysis showed increased fluorescence of the biosensor treated with vanillin at a concentration as low as 200 μM. The average cell fluorescence increases dynamically with an increase in vanillin concentration, with a maximum ~4.5-fold increase observed at 5.0 mM (Fig. 2). Further increases in vanillin concentration adversely affect cell growth due to toxicity. The observed broadening of peaks in the 0.2–3 mM samples may reflect the presence of mixed populations that comprise cells with different levels of vGFP expression, while in the 5 mM sample all cells have shifted to an “on” state with optimum expression of the vGFP protein. Increased fluorescence in the cells treated with 5.0 mM vanillin was clearly visualized by fluorescence imaging (Fig. 3), with mean fluorescence intensity being ~3.5-fold higher than that of untreated cells.

### Specificity of VSC biosensor

**Fig. 4:**
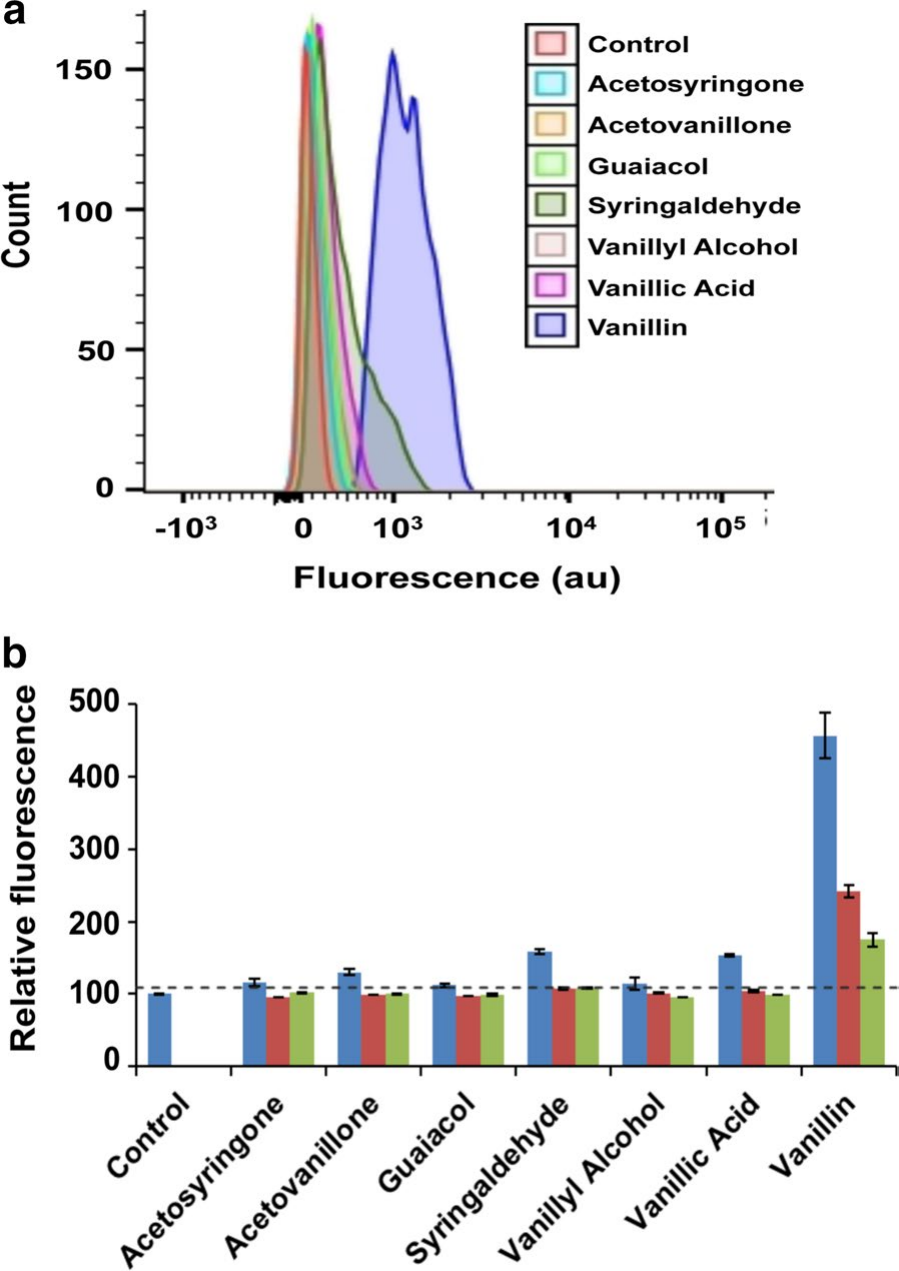
Response of the VSC biosensor to various lignin degradation products: **a** histogram of FACS-generated fluorescence measurement and **b** median fluorescence value (normalized) of the cells treated with 5.0 mM (*blue*), 1.0 mM (*red*) and 0.5 mM (*green*) of individual lignin degradation products. The *dotted line* indicates the significance threshold (fluorescence of the untreated cells +3 SD)

**Fig. 5:**
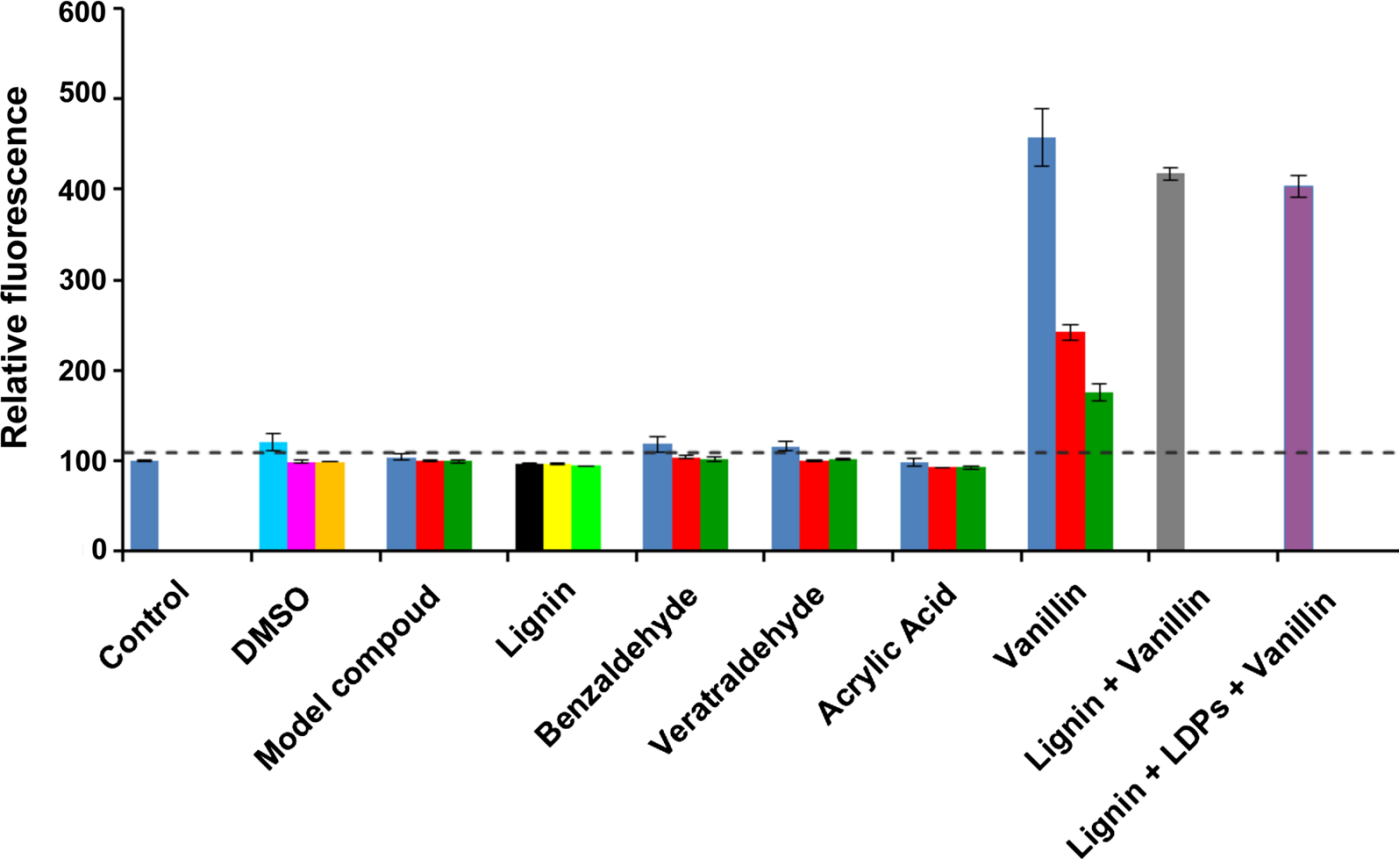
Response of the VSC biosensor to the panel of non-specific chemicals. For model compound, benzaldehyde, veratraldehyde and acrylic acid, treatment with 5.0, 1.0 and 0.5 mM is represented by *blue*, *red* and *green bars*, respectively. For DMSO, treatment with 5.0% (v/v), 1.0% (v/v) and 0.5% (v/v) is represented by *cyan*, *magenta* and *orange bars*, respectively. For lignin, treatment with 5.0, 1.0 and 0.5 mg/ml is represented by *black*, *yellow* and *light green bars*, respectively. For (lignin + vanillin), treatment comprised 5% lignin + 5 mM vanillin and is represented by *grey bar*. For (lignin + LDPs + vanillin), treatment comprised 5% lignin + 5 mM each of acetosyringone, acetovanillone, guaiacol, syringaldehyde, vanillyl alcohol and vanillin, and is represented by *purple bar*. The figure shows median fluorescence value (normalized) of the populations in each sample. The *dotted line* indicates the significance threshold (fluorescence of the untreated cells + 3 SD). LDPs = Lignin Degradation Products

The VSC biosensor was next assayed against various lignin degradation products and kraft lignin that would be present in lignin-degrading reaction systems, along with a dimeric lignin model compound that is often used as a substrate to test potential lignin-degrading properties of enzymes. Cross-reactivity was also tested against DMSO, benzaldehyde and acrylic acid to rule out fluorescence enhancement by solvent, non-specific aromatic aldehydes and a non-specific toxic chemical, respectively. The VSC biosensor does not show any significant response to high concentrations of other potential lignin degradation products (Fig. 4) or non-specific chemicals (Fig. 5) with the exception of syringaldehyde and vanillic acid, both showing ~1.6-fold fluorescence enhancement compared to a 4.5-fold fluorescence enhancement by vanillin. This cross-reactivity may be related to the structural similarity of these compounds with vanillin. No significant fluorescence enhancement was noticed when the biosensor was treated with DMSO, which is used to dissolve lignin and lignin degradation products. No cross-reactivity was observed when the cells were treated with lignin or dimeric lignin model compound (guaiacylglycerol-beta-guaiacyl ether) that is often used to study enzymatic lignin degradation [11, 12, 15]. Response of the biosensor to 5 mM vanillin was not abruptly affected by the presence of 5 mg/ml alkaline kraft lignin alone or in combination with 5 mM each of acetosyringone, acetovanillone, guaiacol, syringaldehyde and vanillyl alcohol. However, about 5–10% less fluorescence was observed when the sensor was treated with the mixtures in comparison to treatment with vanillin alone (Fig. 5). Fluorescence of the VSC biosensor did not change upon treatment with 5 mM acrylic acid, a chemical toxic to *E. coli* [37]. This observation disfavours fluorescent enhancement by any non-specific toxicity-induced overexpression of the vGFP gene. Fluorescence imaging of the biosensor treated with acrylic acid, guaiacol and lignin also showed no fluorescence enhancement, which confirms no cross-reactivity of the sensor with these chemicals (Fig. 3).

### Toxicity of potential lignin degradation products to VSC biosensor

**Fig. 6:**
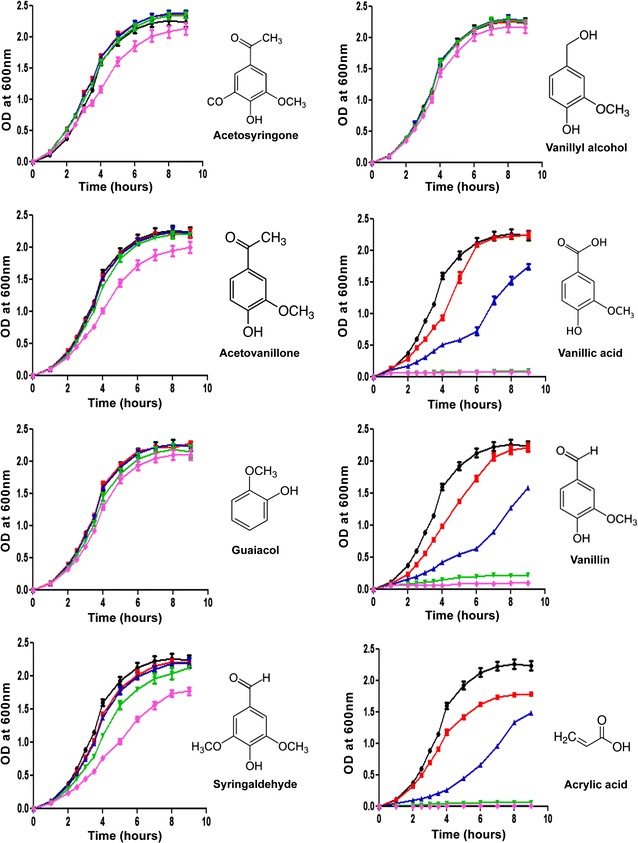
Toxicity of potential lignin degradation products and acrylic acid towards the VSC biosensor. Toxicity was tested with 0 mM (*black line*), 2.5 mM (*red line*), 5.0 mM (*blue line*), 10.0 mM (*green line*) and 20 mM (*magenta line*) of indicated chemicals. The biosensing cells were grown in the presence of individual chemicals and cell growth (OD_600_) was measured at 30-min or 1-h intervals

The toxicity of various lignin degradation products towards the biosensing cells was studied to understand potential inhibition of cell growth with successful lignin degradation (Fig. 6). Although sublethal induction is the basis of vanillin-induced fluorescence enhancement of the VSC biosensor, it would not be able to detect positive mutants developed by directed evolution if high toxicity of any lignin degradation product suppresses cell growth. With the exception of vanillin and vanillic acid, all the major lignin degradation products showed minimal toxicity to the biosensor when treated with up to 20 mM concentrations. Only a slight inhibition was observed at high concentrations (20 mM) of syringaldehyde. As expected, vanillin inhibited the growth of the biosensor at 5 mM concentration but the cells recovered from initial toxicity and entered log phase after 6 h. However, the biosensing cells could not recover within the study time (9 h) from growth inhibition at vanillin concentrations of 10 mM or higher. The toxicity of vanillic acid was very similar to that of vanillin, in agreement with other studies on the antimicrobial activity of vanillin and vanillic acid on *E. coli* [35, 38, 39]. A previous study also showed the complete suppression of *E. coli* growth in the presence of 15 mM vanillin [35]. Friedman et al. [39] have shown that phenolic benzaldehyde and benzoic acid compounds have significant antimicrobial activity against *E. coli*, whereas benzoic acid ester does not. This observation also explains the inhibition of *E. coli* growth by vanillin, syringaldehyde and vanillic acid but not by guaiacol, acetovanillone or acetosyringone. Davidson and Naidu reported that the antimicrobial activity of a phenolic compound depends mainly on its chemical structure and concentration, which supports our observations [40].

We also studied the toxicity of various concentrations of acrylic acid, a non-specific chemical known to be toxic to *E. coli* [37]. Growth inhibition of the VSC biosensor by acrylic acid was similar to that observed with vanillin; it showed substantial toxicity at 5 mM concentration and no growth was observed at higher concentrations.

## Discussion

Lignin can potentially be converted to valuable aromatics such as vanillin, by controlled enzymatic catalysis. While natural enzymes have great potential, their performance could be further improved using directed evolution approaches. In addition to other enzymes, several natural and engineered laccases and peroxidases have been studied for enzymatic conversion of lignin to vanillin [11, 14, 41]. In spite of vast research in this area, no single enzyme has been reported to convert actual lignin substrates to their monomeric phenolic subunits. Remarkably, there are very few reports of selecting engineered enzymes using genuine lignin substrates. This is partially due to unavailability of high-throughput screening tools to detect lignin degradation and also because lignins have extremely heterogeneous structures with versatile chemical linkages that are least likely to be cleaved by the action of a single enzyme [13, 42]. Considering the complexity of lignin structures, future research may be directed towards simultaneous evolution of multiple enzymes or a multi-enzyme pathway using high-throughput screening tools like the live cell vanillin sensor described here. In this respect, the multi-enzyme system described by Reiter et al. [15] is particularly applicable. It was possible to release a small amount of lignin monomers from complex lignin structures using a combination of Cα-dehydrogenase, β-etherase and glutathione lyase enzymes. Salvachua et al. [22] reported lignin depolymerization by fungal secretomes containing a high level of laccase and peroxidase enzymes.

The VSC biosensor described here will be a useful tool in selecting vanillin-synthesizing enzymes from both metagenomic and mutant libraries. Developing enzymes for the conversion of lignin to vanillin would be of particular interest as vanillin is the most important lignin degradation product due to its large-scale use in the food, flavour and cosmetic industries. Induction of the putative *E. coli* promoter used in our biosensor is vanillin specific and no vanillin analogue or non-specific toxic chemical (like acrylic acid) can induce the expression of the vGFP gene under the control of this promoter, which is particularly interesting considering the absence of a known vanillin metabolism pathway in native *E. coli* [43]. However, vanillin’s mode of antimicrobial activity may explain this ambiguity. This comes mainly from its ability to damage the plasma membrane of the microbial cells through interaction with the lipids or proteins, which cause subsequent loss of the ionic gradient across the membrane and inhibition of bacterial respiration [35, 44]. A study using propidium iodide staining suggests that a significant proportion of *E. coli* cells remain alive even after treatment with 50 mM vanillin, although vanillin can completely arrest *E. coli* growth at a concentration of 15 mM, indicating that microbial growth inhibition by vanillin is bacteriostatic in nature rather than bactericidal [35]. This report also showed that *E. coli* can maintain partial potassium gradients after exposure to 50 mM vanillin for 40 min; vanillin treatment in this condition completely dissipates potassium ion gradients of *Lactobacillus plantarum*. Collectively, these observations suggest that the extent of *E. coli* membrane damage caused by vanillin is relatively less severe, and that when exposed to sublethal concentrations of vanillin, *E. coli* may cope with the stress by reestablishing ion gradients by alternative means, without vanillin metabolism. Although we cannot establish any functional group in the up-regulated genes identified by RNAseq analysis of vanillin-treated *E. coli* cells, the functions of the top seven up-regulated genes imply association with osmoprotection, metal ion transport and heavy metal toxicity (Additional file 1: Table S1). The up-regulated gene *ydcI* encodes a putative LysR-type DNA-binding transcriptional regulator. The exact function of ydcI protein is not known yet but other members of LysR-type transcriptional regulators are involved in the expression of various unrelated proteins including sodium–hydrogen antiporter and proteins involved in zinc homeostasis and oxidative stress defence [45, 46]. The proteins encoded by the other two up-regulated genes *yeiW* and *sodC* also play some role in metal ion detoxification and oxidative stress defence. The fourth highest up-regulated gene *proA* encodes a subunit of glutamate-5-semialdehyde dehydrogenase and gamma-glutamyl kinase-GP-reductase multi-enzyme complex that catalyses the first step in the synthesis of the osmoprotective amino acid proline [47, 48].

The VSC biosensor responds in a dose-dependent manner and upon induction with 5.0 mM vanillin fluorescence of the sensor is increased more than 4-fold but further increase of signal is not possible as the *E. coli* cannot grow at higher vanillin concentrations. Detectability within a relatively narrow range of vanillin concentration may be a limitation in selecting for mutants that can produce very low (<0.5 mM) or very high (>5 mM) concentrations of vanillin. Transposing the genetic sensing construct into a vanillin-tolerant microorganism could potentially address toxicity issues. In this respect, the top seven up-regulated genes upon vanillin exposure of *E. coli* cells are conserved within members of the Enterobacteriaceae family including several strains from the genus *Escherichia*, *Shigella*, *Salmonella* and *Enterobacter*. The large molecular weight of lignin precludes ready uptake into microbial cells. The VSC biosensor will likely find optimal use in screening extracellular enzymes that convert lignin to vanillin (Fig. 1). Selection experiments could be carried out on lignin-containing agar plates or using emulsion encapsulation methodology [49]. Additionally, this system could find use in screening enzymes that produce vanillin from smaller cell-permeable precursors. Examples include vanillyl alcohol oxidase and carboxylic acid reductase that produce vanillin from precursors like creosol, vanillylamine and vanillic acid [50, 51]. Versatility of live cell biosensors is also restricted by relatively short cellular lifespans, requirement of specific conditions for growth and survival of microbial systems and restriction of using engineered or live microorganisms in various end products. However, these issues will not create any major challenge in using this biosensor as a host cell for screening lignin-degrading enzyme libraries.

Inducible regulator-based whole-cell sensing systems are established as valuable analytical tools, which allow highly specific detection of target chemicals using fluorescent or bioluminescent reporters [32, 52–56]. While most of these biosensors were developed for environmental microbiology or bioremediation applications, inducible promoter-based sensing systems could be extremely useful in metabolic engineering applications including high-throughput screening of engineered enzyme libraries. Despite their vast potential, only a handful of small-molecule-inducible regulators (e.g. *LacI*) are well characterized and repeatedly used for a diverse range of applications. Development of additional inducible regulators can potentially provide newer biotechnology tools with innovative applications. The product inhibition approach used here could be potentially applied to identify putative promoter regions and develop biosensors for any chemical that exhibits partial toxicity to a microorganism with known genome sequence. Using a similar approach, Rogers et al. [32] developed four genetically encoded biosensors that respond to acrylate, glucarate, erythromycin and naringenin, and demonstrated the usage of glucarate biosensor for selecting superior enzyme variants from the glucarate biosynthesis pathway.

## Conclusions

Enzymatic valorization of lignin will most likely be achieved through engineering of multi-enzyme pathways using a directed evolution approach combined with an effective high-throughput screening technique. The VSC biosensor can provide such a tool, detecting as low as 200 µM vanillin by FACS. This biosensor does not show any cross-reactivity to lignin, vanillin analogues or non-specific toxic chemicals. No major lignin degradation product showed significant toxicity towards the biosensor when treated with up to 20 mM concentration. We propose the use of this biosensor as a host cell for screening of lignin-degrading enzymes from randomized libraries and metagenomic samples.

## Additional file

**Additional file 1: Figure S1.** Determination of sub-lethal dose of vanillin on *E. coli* BL21 cells; **Figure S2.** Cluster heat map showing global gene expression correlation between different sets of samples used in RNAseq experiments; **Figure S3.** Schematic diagram showing location of putative promoter region and the plasmid constructs for development of the whole-cell biosensor; **Table S1.** Top seven up-regulated genes of *E. coli* BL21 strain after exposure to sublethal dose of vanillin and function of the encoded proteins; **Table S2.** Assayed promoter sequences from top seven up-regulated genes; **Table S3.** Fluorescence of the 5 mM vanillin induced live cell biosensors (Lcb1–Lcb7).
