# Supplementary material for: Development of a genetically programed vanillin-sensing bacterium for high-throughput screening of lignin-degrading enzyme libraries
Source: Biotechnol Biofuels. 2017 Feb 3;10:32. doi: 10.1186/s13068-017-0720-5 (PMC5291986; doi:10.1186/s13068-017-0720-5)
Supplement: Supplementary file 1 — Additional file 1: Figure S1. Determination of sub-lethal dose of vanillin on E. coli BL21 cells; Figure S2. Cluster heat map showing global gene expression correlation between different sets of samples used in RNAseq experiments; Figure S3. Schematic diagram showing location of putative promoter region and the plasmid constructs for development of the whole-cell biosensor; Table S1. Top seven up-regulated genes of E. coli BL21 strain after exposure to sublethal dose of vanillin and function of the encoded proteins; Table S2. Assayed promoter sequences from top seven up-regulated genes; Table S3. Fluorescence of the 5 mM vanillin induced live cell biosensors (Lcb1–Lcb7). [file 13068_2017_720_MOESM1_ESM.pdf]

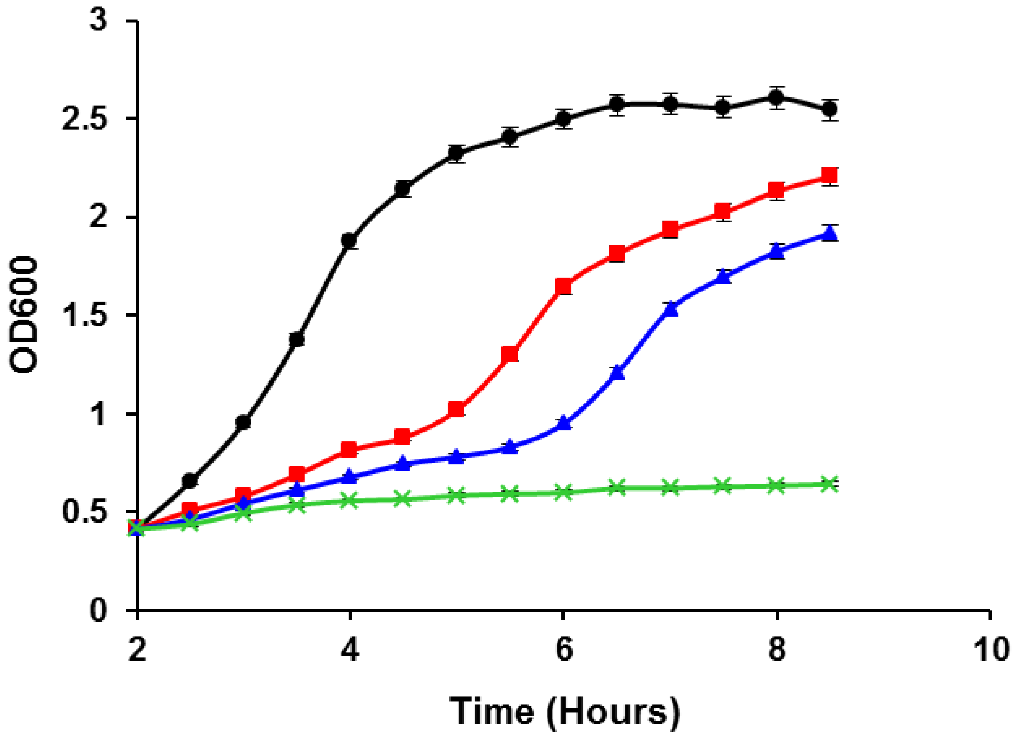

Figure S1. Determination of sub-lethal dose of vanillin on *E. coli* BL21 cells. The cells were grown in LB media containing 0.1 mg/ml ampicillin up to OD_{600} = 0.5 followed by addition of different concentrations of vanillin and microbial growth was monitored by measuring the OD_{600} at 30 minute intervals. The vanillin concentrations studied are 0 mM (black circle), 5 mM (red square), 7 mM (blue triangle) and 10 mM (green cross).

Sample color key

Timepoint	Class	Colour
0 hr	Control (C)	Red
	Vanillin treated (V)	Light red
1 hr	Control (C)	Blue
	Vanillin treated (V)	Light blue
2 hr	Control (C)	Green
	Vanillin treated (V)	Light green
3 hr	Control (C)	Black
	Vanillin treated (V)	Grey

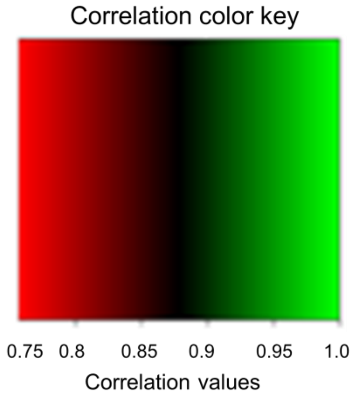

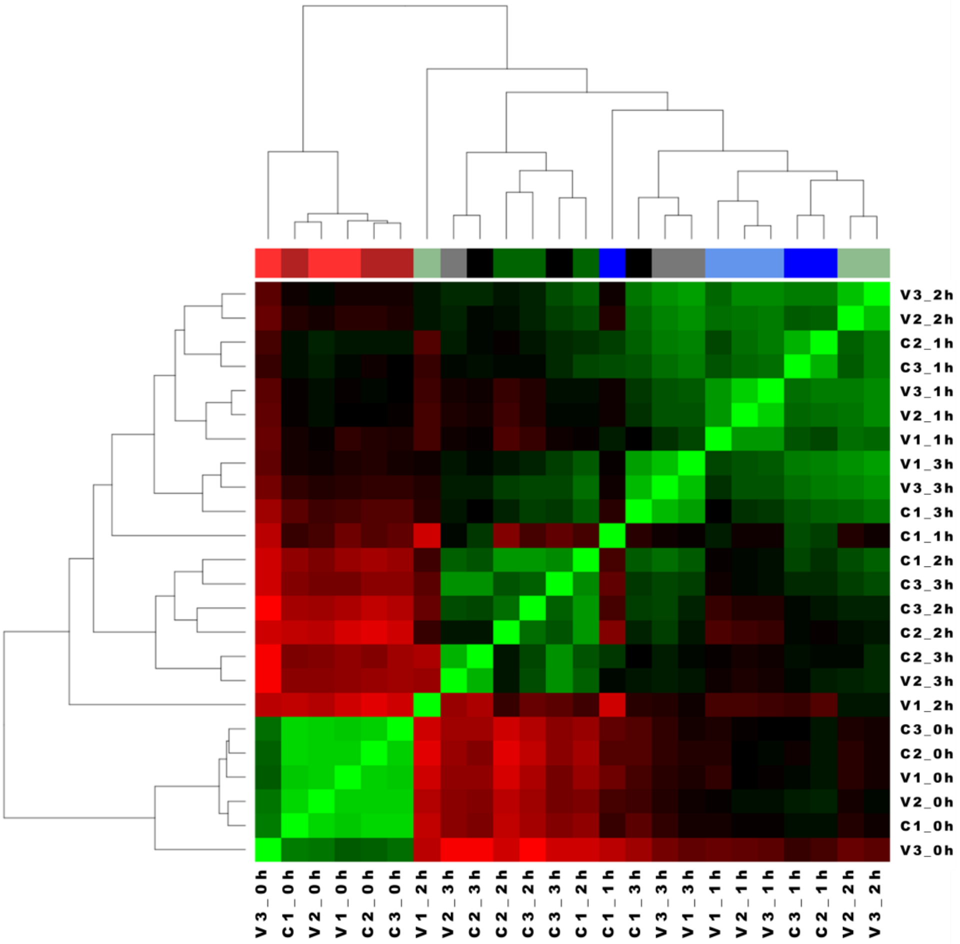

Figure S2. Cluster heat map showing global gene expression correlation between different sets of samples used in RNAseq experiments. The data shows significant variation in global expression profiles in response to vanillin treatment. C or V stands for 'control' and 'vanillin treated' sample respectively. The following number (1, 2 or 3) indicate sample number of the triplicate, and 0h, 1h, 2h and 3h indicate the sampling time points. The hierarchical clustering

was performed using Spearman correlation coefficients of log2 on the FPKM expression matrix in R v3.1.0.

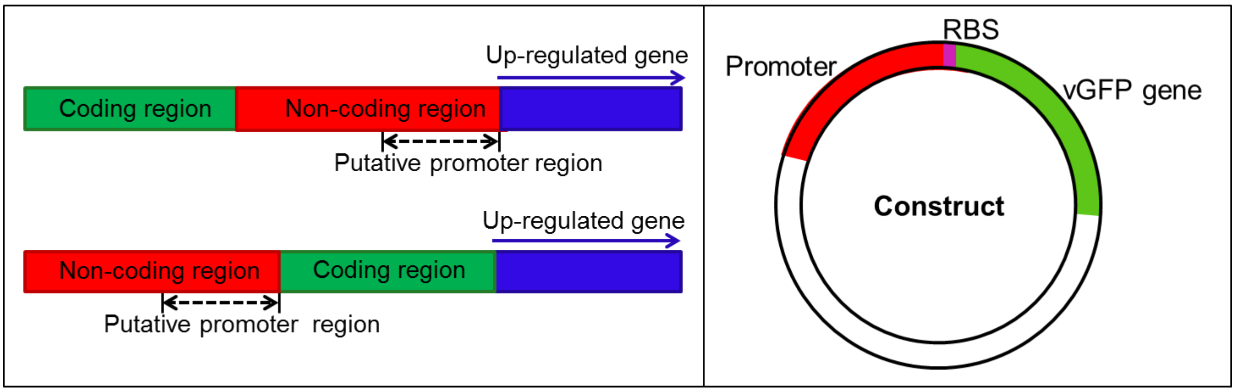

Figure S3. Schematic diagram showing location of putative promoter region (left panel) and the plasmid constructs for development of the whole-cell biosensor (right panel).

Table S1. Top seven up-regulated genes of *E. coli* BL21 strain after exposure to sublethal dose of vanillin and function of the encoded proteins.

**Serial number**	**Gene name**	**Upregulation fold**	**Encoded protein/ Function**
**1**	*yjhD*	15	Hypothetical protein: Function unknown
**2**	*yijF*	12	DUF1287 family protein. Function Unknown
**3**	*ydcI*	11	LysR-type DNA-binding transcriptional regulator.
**4**	*proA*	11	Subunit of glutamate-5-semialdehyde dehydrogenase and gamma-glutamyl kinase-GP-reductase multienzyme complex. Catalyzes the first step in the proline synthesis.
**5**	*yeiW*	9	UPF0153 cysteine cluster protein. Putative zinc- or iron-chelating domain: pfam03692 family.
**6**	*higA*	6.5	Antitoxin of the HigB-HigA toxin-antitoxin system.
**7**	*sodC*	6	Superoxide dismutase precursor. Putative Cu, Zn detoxification.

Table S2. Assayed promoter sequences from top seven up-regulated genes.

Promoter 1 (yjhD):

atccatagctttcgcggcatctgccaccgtgtatttctggtcaacaaccagttgagcggattcgcgtt
taaactctgcgctgaaatttctttttttcattggagcacctgtgttgttctgaggtgagcatatcacc
tctgttcaggtggccaaattcagtgtgccacttcagccctgattcgacttttacaatgacttatctgg
acgcatgaagaacgtcaggaatataccaataacaagcaatgcgacggaaccgtagaatggtaaactcc
agttgcctgttttgtcgataata

Promoter 2 (yijF):

Cctactccaaactcccggcttgtccgggagtttgaacgcaaaattgcctgatgcgctacgcttatcag
gcctacgcaatctctgcaatatattgaatttgcgtgcttttgtaggccggataaggcgttcactccgc
atccggcactctgtgccagtaatctgaaactcccacgttgttcaggaatttttatccgcttctggac
attttctctacagaacaatcgttagcccggaaggtcgaatctgtgactcttattttcacggacgaacc
ag

Promoter 3 (ydcI):

agtaatctggagtgtaggtaaccgcattcactctggcgggaagaatttacaaactgtgatctcgccgc
gaaaacatcaatattatccattttgctgtaacataattgctttaattgttaataatatttgcaatca
agttatcataatcaaacaacttcacttgtcagcgacaccgcttcgtttttaacatcgctt

Promoter 4 (proA):

aacgcgcgaatattcagcgggagagtcccgttgaaaacaggaaagtttttaacctgagattgttaaag
atatattacagattaataatattcttaaaatgtggtaatttagtaaatctgtaataaaagcgtaaaca
actgccgctaggcttgctgatcccgcgcaacaaaacgccatgctttgctcgcagatggttggcaaccg
acgacagtcctgctaaaacgttcgtttgatatcatttttcctaaaattgaatggcagagaatc

Promoter 5 (yeiW):

cgaaaaattcctgtagtcgaaaatgtcaaaaatatcgcgacaaagtactcgcgacgggcctttcgggc
aagtggtattcgcacttttgctggtgcaaaaaaggtggtactgtgcgcgctcatcaatccggtggtta
accttaagagaacaacgt

Promoter 6 (higA):

gattgggagttacctgccggatgcggcgtaaacgccttatccggcctacgttcagcacgttactccag
gccggataagacgcggcaagcgtcgcatccggcaatgtctgcccagttctggaatcgcgcttccataa
aacaaaaaatccctgtcaccatcgtctacattctcttgtttagcgtttttctacgtttattcttccgt
cacacagataaattcatccgttgccaatctggcaacggatgttatcatattgccaatttgtcaacgga
cgtgatgaaga

Promoter 7 (sodC):

ggtgaatccacgttgcaggcaaagttgctcgcccgtcgccaggagatgttcgcgagtatcatgttcgg
tgtgtttgttcatgcgttggagtgtagtagaccagtcggtctagtgcaagcaaagttgctactttgga
cattaacagcttcaataatggtaataatcttatctgaggagggcgaa

Table S3. Fluorescence of the 5 mM vanillin induced live cell biosensors (Lcb1 – Lcb7); the fluorescence was measured using 100 µl overnight-induced cell suspension (OD_{600} = 1.0)

**Sensor**	**Fluorescence**	**Standard deviation**
Lcb1	13,570	711
Lcb2	12,400	1050
Lcb3	10,740	563
Lcb4	430,020	55682
Lcb5	174,050	13854
Lcb6	19,700	1919
Lcb7	15,640	1894
E. coli	105	18
